# Supplementary material for: Content-rich biological network constructed by mining PubMed abstracts
Source: BMC Bioinformatics. 2004 Oct 8;5:147. doi: 10.1186/1471-2105-5-147 (PMC528731; doi:10.1186/1471-2105-5-147)
Supplement: Additional File 2 — The original results of the above study (non-essential files are deleted to keep the file size under the limit set by BMC bioinformatics). [file 1471-2105-5-147-S2.bz2 › chilibotAdditionalFile2/dip40/185ID7526154E627/html/IFNAR1_TYK2.html]

 


 **IFNAR1** and **TYK2** 
  
Found 47 abstracts in PubMed, retrieved 40.  
 

 What does Google say? 
 PDF only 
| .edu only 

---

**Interactive relationship** (e.g. stimulation, inhibition, etc)

**Inhibitory relationship**- Kinase deficient forms of Jak1 and  **Tyk2**  inhibit interferon alpha  [ **IFNAR1** ]  signaling in a dominant manner.  Ref: 9249040 Eur J Biochem, 1997
- Here, it is shown that mutant cells lacking  **Tyk2**  have significantly reduced IFN alpha receptor 1  **IFNAR1**  protein level.  Ref: 9342324 Proc Natl Acad Sci U S A, 1997
- **TYK2**  amino acids 1 601 act in a dominant manner to inhibit the transcription of an interferon alpha  [ **IFNAR1** ]  dependent reporter gene, presumably by displacing endogenous  **TYK2**  from the receptor.  Ref: 9461596 J Biol Chem, 1998
- Signaling by interferon alpha  [ **IFNAR1** ]  IFN alpha, an extracellular factor that mediates a number of anti viral and growth suppressive effects, requires two members of the Janus family of tyrosine kinases JAK family Jak1 and  **Tyk2** .  Ref: 9249040 Eur J Biochem, 1997
**Neutral relationship**- Thus, the major interaction surface lies within JH7 6, but additional JH regions JH5 4 3 contribute in a specific manner to the in vivo assembly of  **Tyk2**  and  **IFNAR1** .  Ref: 9733772 J Biol Chem, 1998
- The tyrosine kinase  **Tyk2**  controls  **IFNAR1**  cell surface expression.  Ref: 12554654 EMBO J, 2003
- Here, we sought to define the molecular basis of the interaction between  **Tyk2**  and  **IFNAR1** , a component of the interferon alpha  [ **IFNAR1** ]  beta receptor, by delimiting a minimal  **IFNAR1**  binding region in the  **Tyk2**  protein.  Ref: 9733772 J Biol Chem, 1998
- Molecular characterization of an alpha interferon receptor 1  [ **IFNAR1** ]  subunit  **IFNaR1**  domain required for  **TYK2**  binding and signal transduction.  Ref: 8628273 Mol Cell Biol, 1996
- Importantly, we demonstrate that  **Tyk2**  slows down  **IFNAR1**  degradation.  Ref: 12554654 EMBO J, 2003
- In the type I interferon receptor,  **Tyk2**  associates with the  **IFNAR1**  receptor subunit and positively influences ligand binding to the receptor complex.  Ref: 12554654 EMBO J, 2003
- Distinct domains of the protein tyrosine kinase  **tyk2**  required for binding of interferon alpha  [ **IFNAR1** ]  beta and for signal transduction.  Ref: 7531704 J Biol Chem, 1995
- The E6  **Tyk2**  interaction requires the JH6 JH7 domains of  **Tyk2** , which are important for  **Tyk2**  binding to the cytoplasmic portion of IFN alpha receptor 1  **IFNAR1** .  Ref: 10523853 Oncogene, 1999
- The human papilloma virus HPV 18 E6 oncoprotein physically associates with  **Tyk2**  and impairs Jak STAT activation by interferon alpha  [ **IFNAR1** ] .  Ref: 10523853 Oncogene, 1999
- This report defines the  **IFNaR1**  interaction domain on  **TYK2** .  Ref: 9461596 J Biol Chem, 1998
- Expression of the N terminal region of  **Tyk2**  in these cells reconstituted wild type  **IFNAR1**  level, but did not restore the binding activity of the receptor.  Ref: 9342324 Proc Natl Acad Sci U S A, 1997
- It was previously shown that  **Tyk2**  contributes to interferon alpha  [ **IFNAR1** ]  IFN alpha signaling not only catalytically, but also as an essential intracellular component of the receptor complex, being required for high affinity binding of IFN alpha.  Ref: 9342324 Proc Natl Acad Sci U S A, 1997
- Here, we report that  **Tyk2**  is essential for stable cell surface expression of  **IFNAR1** .  Ref: 12554654 EMBO J, 2003
- The amino terminal region of  **Tyk2**  sustains the level of interferon alpha receptor  [ **IFNAR1** ]  1, a component of the interferon alpha  [ **IFNAR1** ]  beta receptor.  Ref: 9342324 Proc Natl Acad Sci U S A, 1997
- the different domains of  **tyk2**  have distinct functions, all essential for full interferon alpha  [ **IFNAR1** ]  and beta binding and signaling.  Ref: 7531704 J Biol Chem, 1995
- Our results indicate that catalytically active  **TYK2**  is required for IFN beta mediated tyrosine phosphorylation of STAT3 and IFNAR 1  [ **IFNAR1** ]  in intact cells.  Ref: 10542297 J Biol Chem, 1999
- Glutathione S transferase  **TYK2**  fusion proteins approximating either the JH6 or JH3 domain affinity precipitate  **IFNaR1** , suggesting that these are major sites of interaction within the larger binding domain.  Ref: 9461596 J Biol Chem, 1998
- To address the question of the role of the  **Tyk2**  nuclear pool in interferon alpha  [ **IFNAR1** ]  beta induced biological effects, cells expressing a membrane targeted form of  **Tyk2**  green fluorescent protein were analyzed for their interferon alpha  [ **IFNAR1** ]  responses.  Ref: 11399767 J Biol Chem, 2001
- Catalytically active  **TYK2**  is essential for interferon beta mediated phosphorylation of STAT3 and interferon alpha receptor  [ **IFNAR1** ]  1 IFNAR 1  [ **IFNAR1** ]  but not for activation of phosphoinositol 3 kinase.  Ref: 10542297 J Biol Chem, 1999
- **Tyk2**  is a member of this family that is involved in the interferon alpha  [ **IFNAR1** ]  beta and interleukin 12 signaling pathways via its specific interaction with the  **IFNAR1**  and the beta1 receptor subunits.  Ref: 11399767 J Biol Chem, 2001
- Further, introduction of dimerized glutathione S transferase  **IFNaR1**  fusion proteins into permeabilized cells is sufficient to induce phosphorylation of  **TYK2**  and the receptor, confirming the role of the binding domain in IFNalpha signal transduction.  Ref: 8628273 Mol Cell Biol, 1996
- However, a dose dependent interference with signal transduction via the endogenous Ifnar  [ **IFNAR1** ]  complex was found for STAT1, STAT2, STAT3,  **Tyk2** , and Jak1 activation, for gene induction, and for antiviral activity.  Ref: 11602573 J Biol Chem, 2001
- Two receptors,  **IFNaR1**  and IFNaR2, associate exclusively in a constitutive manner with two JAK proteins,  **TYK2**  and JAK1, respectively.  Ref: 9461596 J Biol Chem, 1998
- Binding of interferon alpha  [ **IFNAR1** ]  IFN alpha to the multisubunit type I IFN receptor IFNR induces activation of the Tyk 2  [ **TYK2** ]  and Jak 1 kinases and tyrosine phosphorylation of multiple signaling elements, including the Stat proteins that form the ISGF3 alpha complex.  Ref: 7559568 J Biol Chem, 1995
- When expressed in  **Tyk2**  negative cells, the JH7 6 region was unable to stabilize  **IFNAR1**  protein levels, a critical function that we previously attributed to the N region amino acids 1 591 of  **Tyk2** .  Ref: 9733772 J Biol Chem, 1998
- **Tyk2**  and JAK1, members of the Janus kinase JAK family of protein tyrosine kinases, are required for interferon alpha  [ **IFNAR1** ]  beta binding and signaling.  Ref: 8702790 J Biol Chem, 1996
- Interferon alpha  [ **IFNAR1** ]  signaling promotes nucleus to cytoplasmic redistribution of p95Vav, and formation of a multisubunit complex involving Vav, Ku80, and  **Tyk2** .  Ref: 10673353 Biochem Biophys Res Commun, 2000
- Interferon alpha  [ **IFNAR1** ]  induces the rapid tyrosine phosphorylation of a number of molecules, including the cognate receptors, JAK family kinases Jak1 and  **tyk2** , and latent transcription factors STATs 1 and 2.  Ref: 8700538 Oncogene, 1996
- In vitro binding studies demonstrate that the amino terminal half of  **TYK2** , which is approximately 600 amino acids long and contains JAK homology JH domains 3 7, comprises the maximal binding domain for  **IFNaR1** .  Ref: 9461596 J Biol Chem, 1998
- Interferon alpha  [ **IFNAR1** ]  IFNalpha can activate several members of the signal transducers and activator of transcription STAT transcription factor family, a process that requires the tyrosine kinases Jak1 and  **Tyk2** .  Ref: 10777558 J Biol Chem, 2000
- Conversely, co expressed  **Tyk2**  greatly enhances surface  **IFNAR1**  expression.  Ref: 12554654 EMBO J, 2003
- Binding of interferon alpha  [ **IFNAR1** ]  IFN alpha to its receptor induces activation of the Tyk 2  [ **TYK2** ]  and Jak 1 tyrosine kinases and tyrosine phosphorylation of multiple downstream signaling elements, including the Stat components of the interferon stimulated gene factor 3 ISGF 3.  Ref: 8550573 J Biol Chem, 1996
- AVP  [ **IFNAR1** ]  also stimulates the tyrosine phosphorylation of the nonreceptor tyrosine kinase proline rich tyrosine kinase 2  [ **TYK2** ]  Pyk2 and promotes Src family kinase phosphorylation at Tyr 418, indicative of Src activation.  Ref: 11832328 Am J Physiol Cell Physiol, 2002

**Non-interactive relationship** (e.g. studied together, co-existance, homology, etc.)

- Among elements involved in signal transduction by type I IFNs, the tyrosine kinase  **Tyk2**  but not Jak1, and the ISGF3 transcription factor subunit Stat2 p113 but not Stat1 p91, are found associated with the IFNAR  [ **IFNAR1** ]  protein.  Ref: 7813427 EMBO J, 1994
- Previous studies have demonstrated that  **TYK2**  directly and specifically binds to and tyrosine phosphorylates  **IFNaR1**  in vitro.  Ref: 8628273 Mol Cell Biol, 1996
- These studies also showed that the N terminal region does not directly modulate the basal autophosphorylation activity of  **Tyk2** , but it is required for efficient in vitro  **IFNAR1**  phosphorylation and for rendering the enzyme activatable by IFN alpha.  Ref: 9342324 Proc Natl Acad Sci U S A, 1997
- Studies of mutant  **Tyk2**  forms deleted at the N terminus indicated that the integrity of the N terminal region is required to sustain  **IFNAR1** .  Ref: 9342324 Proc Natl Acad Sci U S A, 1997
- To investigate the role of the KL domain of  **Tyk2**  in interferon alpha  [ **IFNAR1** ]  beta signaling, we transfected a library of  **Tyk2**  cDNAs containing random point mutations in KL into  **Tyk2**  negative cells and selected for loss of function  **Tyk2**  mutants.  Ref: 10908660 Proc Natl Acad Sci U S A, 2000
- Binding of alpha interferon IFNalpha to its receptors induces rapid tyrosine phosphorylation of the receptor subunits  **IFNaR1**  and IFNaR2, the  **TYK2**  and JAK1 tyrosine kinases, and the Stat1 and Stat2 transcription factors.  Ref: 8628273 Mol Cell Biol, 1996
- In murine macrophages, IFN beta induced TLR3 up regulation required  **IFNAR1** , STAT1, and in part IRF 1, but not the Janus kinase Jak family member  **Tyk2** .  Ref: 12672806 J Biol Chem< MedlineTA>J Biol Chem, 1996
- In the absence of  **Tyk2** , mature  **IFNAR1**  is weakly expressed on the cell surface.  Ref: 12554654 EMBO J, 2003
- We now report a detailed analysis of the  **TYK2**  binding domain on the  **IFNaR1**  subunit.  Ref: 8628273 Mol Cell Biol, 1996
- All together, our findings demonstrate that an intact KL domain is essential for the intrinsic catalytic activity of  **Tyk2**  and for the establishment of a high affinity interferon alpha receptor  [ **IFNAR1** ]  complex.  Ref: 10908660 Proc Natl Acad Sci U S A, 2000
- Despite  **Tyk2**  activation, erythropoietin treatment of cells expressing this erythropoietin receptor  **Ifnar1**  chimera did not evoke any detectable IFN type response.  Ref: 11602573 J Biol Chem, 2001
- Specific contribution of  **Tyk2**  JH regions to the binding and the expression of the interferon alpha  [ **IFNAR1** ]  beta receptor component  **IFNAR1** .  Ref: 9733772 J Biol Chem, 1998
- Thus, while these results add  **tyk2**  to a growing list of interferon alpha  [ **IFNAR1** ]  regulated proteins that might be able to suppress tumor formation, the biochemical basis of this activity remains unknown.  Ref: 8642873 Leukemia, 1996
- Interferon alpha  [ **IFNAR1** ]  IFN alpha mediated intracellular signaling is initiated by ligand induced receptor dimerization, tyrosine phosphorylation of the  **Tyk2**  and Jak1 tyrosine kinases, and subsequent phosphorylation of the Stat1 and Stat2 proteins.  Ref: 8910507 J Biol Chem, 1996
- Overall, these results indicate that distinct  **Tyk2**  domains provide different functions to the receptor complex the N terminal region sustains  **IFNAR1**  level.  Ref: 9342324 Proc Natl Acad Sci U S A, 1997
- Genetic and biochemical evidence supports the requirement for  **tyk2**  in interferon alpha  [ **IFNAR1** ]  beta binding and signaling.  Ref: 7531704 J Biol Chem, 1995
- The receptor blockade by mAb 51.44 and 234.28 resulted in the inhibition of IFN alpha2a and IFN beta induced tyrosine phosphorylation of Jak1,  **Tyk2** , Stat1 2 3, and IFNAR 1  [ **IFNAR1** ]  2 and inhibition of IFN stimulated gene factor 3 ISGF3 formation.  Ref: 11096454 J Interferon Cytokine Res, 2000
- Instead of increased phosphorylation of JAK1 and JAK2 in response to G CSF and interferon gamma, and JAK1 and  **Tyk2**  in response to interferon alpha  [ **IFNAR1** ]  beta, we observed only an increase of phosphorylation of  **Tyk2**  in response to all of these cytokines in NFS 60 cells.  Ref: 8919032 Growth Factors, 1996
- First, we used an in vitro binding assay to identify the  **TYK2**  binding motif in  **IFNaR1**  as well as the critical residues within this region.  Ref: 8628273 Mol Cell Biol, 1996
- Precipitation of cross linked material from U937 cells with anti  **TYK2**  sera revealed a 240 kDa complex, which apparently did not contain IFNAR  [ **IFNAR1** ]  and was not present in IFN resistant HEC1B cells.  Ref: 7479825 Proc Natl Acad Sci U S A, 1995
- Evidence is also provided of the lack of specificity of the  **Tyk2**  kinase like and tyrosine kinase domains in interferon alpha  [ **IFNAR1** ]  beta receptor signaling.  Ref: 9733772 J Biol Chem, 1998
- Like the wild type  **Tyk2** , the mutant proteins were able to sustain the level of  **IFNAR1**  receptor protein.  Ref: 10908660 Proc Natl Acad Sci U S A, 2000
- Interferon alpha  [ **IFNAR1** ]  dependent activation of  **Tyk2**  requires phosphorylation of positive regulatory tyrosines by another kinase.  Ref: 8702790 J Biol Chem, 1996
- The basal expression of IFN alpha receptor  **IFNAR1**  and IF NAR2 JAK1 and  **TYK2**  mRNAs was readily detectable, and their expression was not significantly altered by treatment with either IFN alpha or IFN gamma.  Ref: 11097485 J Hepatol, 2000
- A dual role for the kinase like domain of the tyrosine kinase  **Tyk2**  in interferon alpha  [ **IFNAR1** ]  signaling.  Ref: 10908660 Proc Natl Acad Sci U S A, 2000
- In both model systems, titration of the  **Tyk2**  kinase away from the  **Ifnar1**  receptor chain accounts for the observed cross interference.  Ref: 11602573 J Biol Chem, 2001
- However, all four mutants were incapable of restoring high affinity interferon alpha  [ **IFNAR1** ]  binding in  **Tyk2**  negative cells and were also catalytically impaired, even when transiently overexpressed.  Ref: 10908660 Proc Natl Acad Sci U S A, 2000
- Moreover, substitution of the JH7 JH6 domain in JAK1 with that of  **Tyk2**  did not restore  **IFNAR1**  level nor interferon alpha  [ **IFNAR1** ]  signaling in  **Tyk2**  negative cells.  Ref: 9733772 J Biol Chem, 1998
- For example, IFN beta, but not IFN alpha, induces the association of tyrosine phosphorylated receptor components  **ifnar1**  and ifnar2, and has activity in cells lacking the IFN receptor associated, Janus kinase  **tyk2** .  Ref: 9525899 J Biol Chem, 1998
- This same fragment inhibits interferon alpha  [ **IFNAR1** ]  dependent tyrosine phosphorylation of  **TYK2** , STAT1, and STAT2.  Ref: 9461596 J Biol Chem, 1998
- Definition of the interferon alpha receptor  [ **IFNAR1** ]  binding domain on the  **TYK2**  kinase.  Ref: 9461596 J Biol Chem, 1998
- The type I interferon family includes 13 alpha, one omega and one beta subtypes recognized by a complex containing the receptor subunits  **ifnar1**  and ifnar2 and their associated Janus tyrosine kinases,  **Tyk2**  and Jak1.  Ref: 9737924 J Mol Biol, 1998
